# Evaluating the Effectiveness of Clinical Pharmacy Consultations on Nutrition, Physical Activity, and Sleep in Improving Patient-Reported Psychiatric Outcomes for Individuals with Mental Illnesses

**DOI:** 10.3390/pharmacy7010002

**Published:** 2018-12-22

**Authors:** Jennifer Bingham, David R. Axon, Nicole Scovis, Ann M. Taylor

**Affiliations:** 1 SinfoníaRx, Tucson, AZ 85701, USA; NScovis@sinfoniarx.com; 2 College of Pharmacy, University of Arizona, Tucson, AZ 85721, USA; Axon@pharmacy.arizona.edu (D.R.A.); Taylor@pharmacy.arizona.edu (A.M.T.)

**Keywords:** pharmacist, education, Duke, nutrition, exercise, sleep

## Abstract

One fifth of U.S. adults have a current mental illness. Nutrition, physical activity, and sleep are critical to physical health; any related deficiencies may worsen existing mental health conditions. Little is known about the impact of clinical pharmacist assessment and consultation in improving physical and mental health outcomes. The study objective was to determine whether patients’ mental health status improved following clinical pharmacist consultation. This pilot study involved clinical pharmacist-delivered services at an integrated medical behavioral health clinic in June 2018. Inclusion criteria required adults aged 18 years older, an established mental health diagnosis, and taking ≥2 prescribed psychotropic medications. One pharmacist conducted telephonic, medical, and psychiatric health risk assessment and counseling to improve nutrition, physical activity, and sleep status, both initially and at two-week follow-up. The Duke Health Profile (Duke) physical, anxiety, depression, and anxiety-depression scores measured patients’ pre/post changes. Participants (*n* = 20) experienced higher Duke physical scores (*p* = 0.007) and significantly lower anxiety (*p* = 0.025), depression (*p* = 0.001) and anxiety-depression scores (*p* = 0.005) at follow-up. This pilot study provides preliminary evidence for pharmacist-led, targeted, telephonic counseling in improving short-term physical and mental Duke health scores. Further research evaluating the impact of clinical pharmacists’ role in improving physical and behavioral health outcomes is warranted.

## 1. Introduction

Mental health conditions are prevalent in the United States (U.S.), with 55% of the population expected to experience a mental health illness during their lifetime [1]. The 2015 National Survey on Drug Use and Health estimated that roughly one in five U.S. adults had a current mental illness [2]. Of these, approximately 22% (9.7 million) of adults did not receive mental health care [3]. Individuals with mental illness have decreased access to healthcare and monitoring services for physical health conditions [4]. Additionally, the deficit in mental health professionals has forced patients to seek psychiatric care from their primary care providers [3]. Thus, a gap exists in service provisions to address patients’ physical and mental well-being, which is critical to one’s overall health.

The effects of mental illness are far-reaching, affecting many facets of patients’ lives. Specifically, these patients are at increased risk of physical health problems [5], and of experiencing increased morbidity and mortality secondary to preventable conditions with modifiable factors, including alcohol consumption, smoking, poor nutrition, and lack of exercise [4]. Furthermore, an interrelationship exists between psychiatric outcomes and lifestyle habits, such as diet, exercise, and sleep [6,7,8]. Several neurotransmitters (e.g., serotonin and norepinephrine) require intake of essential dietary amino acids, vitamins and adequate sleep for synthesis to occur, as well as physical activity for their release. Inadequate consumption of appropriate foods may worsen anxiety, depression, and insomnia [7,8], while physical activity can reduce their severity [9].

Lifelong preventive care is critical to guide appropriate nutrition, physical activity, and sleep to prevent negative health outcomes, whereas improved health outcomes typically are influenced by positive lifestyle habits. To date, a few studies have investigated the impact of pharmacists’ role in mental health care. Integration of a clinical pharmacist into a collaborative care model in combination with direct, patient-centered counseling and education can improve health outcomes [10,11]. Yet, little is known about pharmacists’ involvement in preventive strategies to improve diet; assess deficiency of essential amino acids, vitamins, and minerals; encourage more physical activity; and recommend adequate daily sleep.

To address this gap in service provision, a clinical pharmacist developed a pilot program to offer preventive mental health care services to patients. The pharmacist assessed patients’ current nutrition, physical activity, and sleep status; subsequent counseling and education were provided as deemed appropriate.

The study objective was to determine whether clinical pharmacist consultation improved patients’ mental health status, measured via the Duke Health Profile (Duke) mental health scales (anxiety, depression, anxiety-depression) two weeks after the clinical pharmacist consultation.

## 2. Materials and Methods

### 2.1. Study Design

This pilot program involved a retrospective record review of patients who participated in a pharmacist-delivered program designed for individuals with mental health conditions; the program was conducted at an integrated medical behavioral health clinic. The program was designed specifically to optimize management of mental health conditions by focusing on patients’ nutritional intake, physical activity level, and sleep quality and quantity. The Institutional Review Board approved this study (protocol number 1804441035, approved 20 April 2018).

### 2.2. Study Sample

Eligible individuals met these study criteria: 18 years of age or older; an established (receiving care for at least a year) patient at the clinic; designation of general mental health condition by the Arizona Health Care Cost Containment System (Arizona’s Medicaid program); and were taking at least two chronic, psychotropic medications (e.g., alpha-2 antagonist, antipsychotics, second-generation atypical antipsychotics, monoamine oxidase inhibitors, non-dopamine reuptake inhibitors, nefazodone, serotonin reuptake inhibitors, selective serotonin reuptake inhibitors, and U.S. Food and Drug Administration (FDA)-labeled mood stabilizers). The sampling method was based on the primary investigator requesting clinic records for patients meeting the study inclusion criteria. Individuals were excluded if they were less than 18 years of age, had developmental disabilities, had a documented inpatient hospitalization within the last 90 days, or were enrolled in an existing wellness program at the clinic. Participants voluntarily enrolled in an existing wellness program at the participating site were excluded to avoid confounding by providing duplicative clinical services.

### 2.3. Initial Consultation (Intervention)

The initial assessment and consultation, utilizing a standardized call script (see Appendix A), was conducted via telephone, and consisted of multiple components. First, the pharmacist reconciled the patient’s medication list, reviewed allergies, assessed adherence to medications, and assessed nutritional intake, physical activity, and sleep quality. Next, the pharmacist provided standardized educational counseling on strategies to improve dietary precursors for targeted neurotransmitters, specific to the patient’s medications’ mechanism(s) of action. The pharmacist then addressed barriers (e.g., access to appropriate food) to physical, nutritional, and sleep adherence recommendations; pharmacist-delivered patient education was based on relevant guidelines [e.g., Recommended Dietary Allowances (RDA), American Academy of Sleep Medicine and Sleep Research Society, Centers for Disease Control and Prevention (for sleep hygiene), Office of Disease Promotion and Health Promotion (for physical activity)] [12,13,14,15]. Finally, the pharmacist identified psychotropic medications’ targeted neurotransmitter(s) (e.g., serotonin, norepinephrine, and dopamine), based on the mechanism of action. This information, in combination with the patient’s status (e.g., chronic conditions, self-reported dietary and supplement intake), assisted the pharmacist in determining the proteins essential for achieving nutritional intake of neurotransmitter precursors (e.g., L-tryptophan for serotonin, phenylalanine for norepinephrine, and dopamine).

### 2.4. Physical and Mental Health Assessment

The Duke instrument assessed patient health status. The 17-item instrument measures general, mental, physical, social, and perceived health, plus anxiety, depression, pain, disability and self-esteem [11]. This study only assessed patient’s physical and mental health. Both high Duke physical scores (0 = poorest health, and 100 = best health) and low anxiety, depression, and anxiety-depression scores (0 = best health, and 100 = poorest health) represented better controlled conditions [16].

Variables included the physical Duke score, anxiety Duke score, depression Duke score, anxiety-depression combined Duke score. The pharmacist reviewed baseline Duke scores prior to the initial consultation; the scores were provided to the pharmacist by the clinic staff who obtained the data from the electronic health record. At the two-week follow up, the pharmacist conducted the assessment with the patient. Follow-up scores were collected from the electronic health record software program that housed a built-in Duke health survey; however, the research team did not have access to these scores in the electronic system. Baseline and follow-up scores were compared to assess changes in physical and mental health status scores, after the pharmacist intervention.

The pharmacist conducted comprehensive reviews, during the intial and follow-up consultations, to address each of the following:

#### 2.4.1. General Nutrition Counseling

- Assess patient dietary and supplement intake of essential nutrients required for optimal absorption, based on patient recall of a typical daily meal plan. Address apparent deficit(s) and make recommendations to supplement patient’s intake of these nutrients.
- Identify strategies (e.g., educate patient on integrating good food sources of essential nutrients or encourage tracking intake of essential nutrients) for achieving the Acceptable Macronutrient Distribution Range of 10–35% of healthy complete protein daily and creating individualized meal plans through open discussion, based on patient dietary restrictions, comorbidities, and financial barriers [12].
- Recommend smaller, more frequent meals throughout the day, when appropriate.
- Ensure alignment with prescribed medications for achieving optimal mental health status.

#### 2.4.2. Serotonin-Targeting Psychotropic Medications

- Assess sources of complete protein, including all nine essential amino acids and L-tryptophan (e.g., meat, poultry, fish, dairy, eggs); sources containing all essential amino acids were deemed necessary for patients taking a serotonin-targeting psychotropic medication [17].
- Evaluate dietary intake of vitamins B6, B9, B12, and D3, as well as omega fatty acids for enzyme activation of tryptophan hydroxylase [17]. In cases where the patient reported dietary intolerance or avoidance of complete proteins, the pharmacist emphasized these precursors.

#### 2.4.3. Norepinephrine and Dopamine-Targeting Psychotropic Medications

- Assess nutritional intake of essential amino acids phenylalanine and tyrosine, as well as vitamins B6, B9, B12, D3, and iron (for dopamine) for tyrosine hydroxylase activation.
- Encourage consumption of incomplete proteins containing phenylalanine and tyrosine (e.g., nuts, grains, beans, legumes, soy, or animal-based complete proteins).

#### 2.4.4. Sleep Counseling

- Assess sleep quality and quantity based on the amount of patient-reported uninterrupted sleep received each night. Patients were advised to sleep at least seven hours per night, according to consensus guidelines [13].
- Educate on sleep hygiene, including the importance of maintaining a consistent routine (e.g., rising and going to bed at same time daily); removing distractions (i.e., computers, electronic devices, televisions, cellular phones); and avoiding large meals, physical activity, and stimulants (e.g., smoking, caffeine) before bedtime, per the Centers for Disease Control and Prevention recommendations [14].

#### 2.4.5. Physical Activity Counseling

- Assess physical activity level. Patients were advised to engage in 150 minutes of physical activity each week per the Office of Disease Prevention and Health Promotion guidelines [15]. A personalized plan was created after the pharmacist and patient mutually agreed on a physical activity schedule that aligned with the patient’s normal routine (e.g., shorter sessions throughout the day if needed).
- Recommend consulting with their healthcare provider before starting a new program, and selecting physical activities of interest to help increase motivation and maintenance.

### 2.5. Follow-up Consultation (Intervention)

During this telephonic consultation, the pharmacist revisited follow-up points from the initial call, assessing changes in nutrition, physical activity, and sleep habits using a standardized call script. The pharmacist also conducted a Duke assessment with the patient.

### 2.6. Data Collection and Statistical Analysis

Data from the clinic’s electronic health record and the Duke instrument were used to assess the impact of the pharmacist interventions on respective physical and mental health. At the beginning of the initial telephone call, the primary investigator (PI) briefly described the study; if the patient chose to participate, he or she provided verbal consent to the PI at that time. Participants also were mailed a waiver of consent document outlining pertinent information related to the research project. Patient confidentiality and anonymity was maintained throughout the study.

The baseline scores were collected in the electronic health record (EHR) by clinic staff and provided to the pharmacist just prior to the initial consultation; the PI/pharmacist did not have access to the patient’s EHR during the study. To minimize bias, the baseline outcome data (i.e., DUKE scores) were entered and stored in Research Data Electronic Capture (REDCap) secure web application (Vanderbilt University Medical Center, Version 8.10.0, Nashville, TN, United States) [18]. The follow-up data was collected initially by the pharmacist in an Excel tracking spreadsheet (Microsoft Office 360 Plus) containing labels (no identifiers). The pharmacist became aware of the relationship between the baseline data (e.g., Duke scores) upon entering the follow-up data in REDCap, after completing the telephonic follow-up consultation. To ensure data security and integrity, the PI was HIPAA-certified and the only person who had access to the final coded dataset. Physical and mental health (anxiety, depression, and anxiety-depression) scores were reported at the initial and follow-up consultations and compared using paired t-tests. An alpha of 0.05 was set a priori. Analyses were conducted using SPSS (IBM Corp., Version 21, Armonk, NY, United States).

## 3. Results

**Figure 1:**
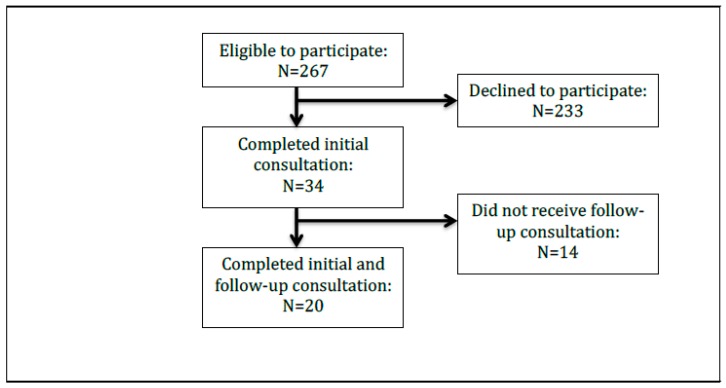
Flowchart showing the number of individuals in the study.

A total of 267 eligible patients met the inclusion criteria for this pilot study. Of these, 34 participated in the program, and 20 (59%) received both the initial and follow-up consultations (see Figure 1). Participants were typically female (60%), white (60%), non-Hispanic or Latino (60%), and younger (less than 40 years old, 60%), although all age groups were well represented in this study. Interestingly, 40% of participants were 31 to 40 years of age, while one fifth were less than 30 years of age. Additionally, seven participants did not report their race nor ethnicity. All participants had Medicaid as both their primary and secondary healthcare insurance. For further details, see Table 1.

There were significant improvements in both the physical and mental health Duke measures. The physical scores increased from baseline to follow-up, indicating improvements in lifestyle habits. Furthermore, significant reductions were observed in the anxiety (*p* < 0.025), depression (*p* < 0.001), and anxiety-depression (*p* < 0.005) scores between initial and follow-up consultations. For further details, see Table 2.

From the initial to follow-up measurement, there was a larger change among females compared to males for depression score (mean change −32.5 ± 26.3 versus −7.5 ± 19.8, respectively; *p* = 0.035) and depression-anxiety score (mean change −31.0 ± 28.0 versus −3.6 ± 21.6 respectively; *p* = 0.031). Likewise, there was a larger change among those reporting their race as white compared to their non-white counterparts for the anxiety score (mean change −25.0 ± 22.5 versus 3.1 ± 19.9 respectively; *p* = 0.010), depression score (mean change −33.3 ± 22.7 versus −6.25 ± 24.5, respectively; *p* = 0.021) and depression-anxiety score (mean change −33.3 ± 24.4 versus 0.1 ± 22.6, respectively; *p* = 0.006).

## 4. Discussion

These pilot study findings provide preliminary evidence to support the value of pharmacist-led consultations for underserved patients with mental health conditions. Specifically, patients with mental health conditions often require additional health care services [4,5], and the pharmacist is uniquely positioned to help address, and ultimately improve those patients’ health outcomes [10].

The pilot program demonstrated a larger change among females versus males for depression scores. This parallels other models that found telephonic consultations improved the management of depression [19].

The majority of participants in this mental health pilot study were female, suggesting that they may be more likely than males to seek [20] and utilize mental health services [21,22]. White, non-Latino individuals represented the majority of patients in this study, which aligns with research reported by others regarding help-seeking behavior for mental health conditions [23]. This may speak to the reluctance of some groups of individuals (e.g., African Americans, non-white Hispanics) to seek mental healthcare services. To that end, African-Americans and Hispanics (e.g., non-white Latinos) may be less likely to perceive the need for help [24]. Additionally, African-Americans are more likely to believe that mental conditions will improve on their own [25], relying more on informal support systems compared to non-Latino whites. Furthermore, minority groups typically underutilize mental health services [26,27,28,29].

With regard to race/ethnicity, the current study demonstrated a larger change in Duke scores among those reporting their race as white compared to non-whites for anxiety, depression, and depression-anxiety. Other factors (e.g., mental health literacy, economic resouces) may partially explain some of these differences in Duke scores. For example, some non-white groups may experience disparities (e.g., limited access and ability to incorporate better food sources of essential amino acids), potentially affecting their response and ability to engage in more positive health behaviors [23].

The telepharmacist was board-certified in ambulatory care pharmacy, experienced in the provision of medication therapy management consultations, and had completed a psychiatric pharmacy certificate program. While this pharmacist had considerable advanced education and training, it is important that appropriate academic and lifelong programs are developed, evaluated, and available to support pharmacists in providing these specialized services. Further research is needed to determine pharmacists’ perceptions regarding their ability to provide counseling services to patients with mental health conditions, and more specifically, after participating in programs designed to address the health care needs of these underserved patients.

Furthermore, the pharmacist’s role was critical in the provision of medication management, and in particular, for psychotropic medications. With added emphasis on lifestyle habits, the pharmacist was key in addressing patients’ needs and providing expertise and medication support in light of the apparent provider shortage. Providing personalized care during the consultations was critical to improved outcomes, yet was quite time-consuming. However, the average time spent on each consultation was not documented for this study. Tracking of the pharmacist-related resources necessary for implementing this integrated model in other clinical settings is warranted in future studies.

Further expansion of the current pilot study is of interest, specifically to include investigation of this populations’ vulnerability to substance abuse. This could provide valuable insight for researchers with regard to these vulnerable individuals. Additionally, this sample population included younger patients with mental health challenges, all of whom were receiving healthcare benefits through Arizona’s managed care Medicaid benefit. However, future work could include implementing this integrated model in a subset of patients with anxiety disorders (e.g., agoraphobia and those susceptible to isolation), to better understand the pharmacist’s role in helping these patients adhere to their medical appointments, and thus improve their health outcomes.

It is critical to build in longer-term patient follow-up to study the effects of the pharmacist’s interventions on self-reported health outcomes. Moreover, integrating an interprofessional coordinator in to programs such as this is vital to implement strategies for increasing patient engagement and reducing attrition (e.g., contacting patients for telephonic pharmacist consultation appointments); to serve as a liaison to enhance patient–provider–pharmacist communication; and to improve overall program efficiency.

While the pharmacist assessed dietary and supplement intake, more accurate measures of consumption are warranted. For instance, a tracking log or diet records could provide insight regarding the exact sources of protein. Utilizing a food frequency questionnaire, designed specifically to measure protein intake, could assist the pharmacist in assessing average consumption over time (e.g., months rather than days) [30], as well as providing valuable information to integrate into the pharmacist’s patient-related recommendations during the consultations. Additionally, the pharmacist’s medication reconciliation was based on patient-reported information, given that EHR was not granted to study personnel. Future work must include capturing this valuable medication data to enrich the reportable results.

Preventive care is an established benefit of physical health care, especially given that diet and exercise are routinely discussed during half of primary care visits [31]. However, less is known about the benefits of preventive care in the behavioral health setting. The results of this study lend evidence to the value of routinely incorporating prevention when caring for individuals with behavioral health needs.

### Limitations

This pilot study had its limitations. First, a substantial number of patients (*n* = 14; 41%) were lost at the follow-up, considerably affecting the sample size. Second, patients were only followed for two weeks before the second measures were taken, preventing the ability to draw long-term conclusions. Third, all data collected was self-reported by the participant, and was therefore susceptible to bias. Fourth, the consultation was conducted via telephone, and presented challenges with monitoring consumption and adherence to interventions. Fifth, the fact that the pilot program did not capture patients’ medications in the dataset is a study limitation; in hindsight, having this information could provide valuable insight. This study was only conducted in one outpatient clinic setting with Medicaid patients, thus limiting its generalizability to other settings and populations.

## 5. Conclusions

This pilot study provides preliminary evidence regarding the benefits of pharmacist-provided lifestyle counseling in improving participants’ Duke physical and mental (anxiety, depression, anxiety-depression) health scores at the two-week follow up. These results highlight the value of the pharmacist’s involvement, suggesting the potential for improved nutrition, physical activity, and sleep for patients with mental health conditions, at least in the short term. However, more research is warranted to determine the impact of similar pharmacist interventions on medical and behavioral outcomes and long-term health benefits, as well as integrating such a concept into accredited pharmacy education programs.

## Figures and Tables

**Table 1:** Demographic characteristics of participants (*N* = 20) participating in a pharmacist-delivered program for Medicaid patients with mental illness.

Characteristic	*N* (%)
**Age (in years)**	
20–30	4 (20)
31–40	8 (40)
41–50	6 (30)
≥51	2 (10)
**Gender**	
Female	12 (60)
Male	8 (40)
**Ethnicity**	
Not Hispanic or Latino	12 (60)
**Race**	
American Indian or Alaska Native	0 (0)
White	12 (60)
Asian	0 (0)
Black or African American	1 (5)
Unknown	7 (35)

**Table 2:** Differences between Duke Health Profile Scores of participants at baseline and follow-up (*n* = 20).

Profile Scale Scores	Baseline Mean (SD)	Follow up Mean (SD)	*p*-value
**Physical**	54.5 (21.9)	74.5 (17.9)	0.007
**Mental**			
Anxiety	49.6 (26.1)	35.8 (17.7)	0.025
Depression	53.5 (31.2)	31.0 (18.3)	0.001
Anxiety-depression scale	49.3 (31.8)	29.3 (18.4)	0.005
**Overall**	50.8 (29.7)	32.0 (18.1)	0.010

**Key**: SD: standard deviation. The level of significance was set at <0.05.

## Appendix A: Call Scripts

**Nutrition**

The pharmacist researcher will reconcile the medication list with the patient. After reviewing their entire medication list, they will identify the specific neurotransmitter that applies to their regimen and follow the appropriate script below:

- Serotonin helps with your mood. It is also what makes you feel happy. Your [medication name] needs serotonin to work.
- Norepinephrine helps with your response to stressful situations. It can also help with depression. Your [medication name] needs norepinephrine to work.
- Dopamine helps with your emotional response and mental focus. Your [medication name] needs dopamine to work.

“I am going to ask you some questions about your diet to see if you are getting enough of the right foods for your body to build [serotonin/norepinephrine/dopamine].”
“What does your daily meal plan look like?” Review with patient. Identify strengths within diet to build positive foundation. Identify/address cost-related barriers.
“How often do you eat? It is recommended to eat every couple of hours during the day. Do not skip meals, particularly breakfast. When you eat, try to eat slowly over 30 minutes. It will allow your body time to absorb the nutrients better.”
“Do you know which foods are classified as complete proteins? The greatest building blocks for [serotonin/norepinephrine] are things that you might already have in your kitchen. You can find them in beef, venison, buffalo, pork, fish, shellfish, cheese, cottage cheese, milk, yogurt, and eggs.”
“Do you know which foods are classified as incomplete proteins? The building blocks for [norepinephrine/dopamine] are also found in your kitchen. In addition to eating complete proteins, it’s also important to eat incomplete proteins as well. You can find these in nuts, grains, beans, legumes, and soy.”
“How many servings of protein do you eat in a day? Now that we know what types of protein you eat, it is important to talk about how much you consume to make sure you are getting enough to allow your medications to work their best.” Review with patient. Identify methods to achieve 3–4 servings of healthy protein daily intake.
“What else is included on your meal plan?” Based on patient’s medication regimen, the pharmacist will follow the appropriate script below:
“When we think about serotonin building blocks, key vitamins play an important role as well. Vitamin B6 is a great nutritional source of this vitamin, and is found in whole grains, vegetables, and nuts. Vitamin B12 is found in meats, fish, liver, and milk. Folic acid and Vitamin D3 are often found in fortified foods like dairy products, orange juice, and yogurt. Omega-3 Fatty Acids are found in fish, dairy products, and grains.”
“When we think about norepinephrine building blocks, key vitamins play an important role as well. Folic acid is often found in fortified foods like dairy products, orange juice, and yogurt. Vitamin B12 is found in meats, fish, liver, and milk.”
“When we think about dopamine building blocks, key vitamins play an important role as well. Vitamin B6 is a great nutritional source of this vitamin, and is found in whole grains, vegetables, and nuts. Folic acid is often found in fortified foods like dairy products, orange juice, and yogurt. Vitamin B12 is found in meats, fish, liver, and milk. Iron is also a key player. It is found in beans, leafy greens, and fortified cereals.”

**Sleep**

“How many hours of sleep do you get on average per night?” Adults 18 to 60 years should sleep at least seven hours per night.
“Do you have trouble with sleeping?” If patient answers yes, ask for more details. Review relevant sleep hygiene tips (online: https://www.cdc.gov/sleep/about_sleep/sleep_hygiene.html).

**Physical Activity**

“How would you describe your physical activity? Being physically active is important not only for heart health, but also for mental health. It is recommended to be physically active for 30 minutes a day, five times a week. Another option is to break it up. If you have a busy schedule, try dividing your time into two or three segments of 10 to 15 minutes per day. Do not start a new exercise program without talking with their doctor first.” Ask the patient what types of physical activity they like to do. Motivational counseling.
